# Gender-related factors associated with delayed diagnosis of tuberculosis in Eastern Europe and Central Asia

**DOI:** 10.1186/s12889-022-14419-8

**Published:** 2022-11-01

**Authors:** Nonna Turusbekova, Cristina Celan, Liliana Caraulan, Oxana Rucsineanu, Mariam Jibuti, Oxana Ibragimova, Nargis Saidova

**Affiliations:** 1 TBC Consult, Antwerp, Belgium; 2 Center for Health Policies and Studies (PAS Center), Crisinau, Moldova; 3 Crisinau, Moldova; 4 NGO SMIT, Balti, Moldova; 5 New Vector, Tbilisi, Georgia; 6 ALE: Kazakhstan Union of People, living with HIV, Almaty , Kazakhstan; 7 NGO: Gender and Development, Dushanbe, Tajikistan

**Keywords:** Tuberculosis, Gender, Diagnosis

## Abstract

Tuberculosis (TB), a preventable and treatable disease, yearly affects millions of people and takes more than a million lives. Recognizing the symptoms and obtaining the correct diagnosis are vital steps towards treatment and cure. How timely a person with TB gets diagnosed may be influenced by biological differences between the sexes, and factors that are linked to the person’s gender, in the context of the prevailing gender norms. According to our hypothesis, gender-related factors contribute to delays in the diagnosis of TB. We investigated four countries (Georgia, Kazakhstan, Republic of Moldova, and Tajikistan) of Eastern Europe and Central Asia (EECA) - a region with a high burden of drug-resistant TB, scarcity of gender-related TB information, and varying gender equality. Retrospective information was collected directly from the people with a history of TB - through in-depth interviews and focus group discussions. We did not find differences between genders in the way participants recognized TB symptoms. In three countries women de-prioritized seeking diagnosis because of their lack of access to finances, and household-related obligations. In all four countries, men, traditionally carrying the weight of economically supporting the family, tended to postpone TB diagnosis. In two countries women experienced stigma more often than men, and it was a deterrent factor to seeking healthcare. The role of gender in obtaining the correct diagnosis came forth only among the respondents from Georgia and to some extent from Kazakhstan. We conclude that there are barriers to health care seeking and TB diagnosis that affect differently women, men and gender-diverse persons in EECA Region.

## Introduction

Globally there are more Tuberculosis (TB) cases notified among men than women [1], however TB case notification is a complex indicator which combines various factors, from symptoms recognition to accessing health care services and obtaining a correct diagnosis [2]. Some of these factors are nested in biological differences between the sexes [2–4], and others are linked to gender and gender norms which, in turn, influence exposure [2], govern behavior and create structural health system differentials that impact the health of men, women, and gender-diverse people.

From the point of view of public health, timely symptoms recognition and diagnosis of TB are the two keys to commencing effective treatment and thus contributing to a reduction of TB transmission. At the person/patient level, timely diagnosis increases the likelihood of a positive treatment outcome, reduces suffering, and brings down financial costs. A TB diagnostic delay is the time from the symptoms onset to identifying that a person has TB; any such interval that is more than a country’s average could be considered a long diagnostic delay. According to the World Health Organization (WHO), around a quarter of the world’s population are infected with *Mycobacterium tuberculosis* [1], under particular circumstances TB infection may progress to TB disease. There may be a particular vulnerability to such progression among women of reproductive age linked with exposure and acquisition of TB infection [3, 5]. While incidence in pregnant individuals does not differ from that of the general population they experience longer diagnostic delays, some of which are due to a reluctance of health care providers to prescribe and of patients to undergo chest X-ray [6].

A total diagnostic delay consists of a patient delay (time from symptoms onset to a consultation with a health care provider) and a provider or health system-related delay (time from the first consultation with the patient to the date of the correct diagnosis). A systematic review and meta-analysis by Getnet et al. [7] showed delays of at least one month, where 42% of patient delay was associated with illiteracy while health system delay was associated with seeking initial care from informal providers. Getnet’s findings are not disaggregated by sex or gender. However, in many settings, women rather than men are found to delay diagnosis [4], particularly by initially turning to informal health care providers [8], or self-medicating [9]. In countries like India, women aged 15–24 years were found to use private providers more than men [4], the authors linked it to stigma and confidentiality concern of women of marriageable age. Similarly, in Bangladesh and Malawi lack of agency, stigma and self-stigma among women were associated with diagnostic delays [10]. Stigma and discrimination, especially in health care settings, are contributing factors that limit this group’s access to health services globally [11].

In a study in Nepal outreach TB case detection yielded 46% of female patients, while clinic-based passive case finding detected only 28% of women with TB from the overall pool of patients [4]. Findings indicating limited access of women to public TB clinics were also made in Bangladesh [12]. Women with respiratory symptoms appeared to be referred to sputum smear examination less often than men [4, 12], although it was not clear whether this was due to atypical symptoms [13], a difference in how women and men experienced and interpreted their symptoms [14] or other reasons. Women have also been noted as having more challenges to produce usable sputum samples [14], because of inability or embarrassment, while inducing sputum and enabling microscopy considerably increased the yield among women [8, 15]. Finally, women’s limited access to child-care, distance to clinic [16] and lack of finance [10, 17, 18] reduced their access to health care facilities and services, and contributed to diagnostic delays.

Most of the cited studies have been carried out in Africa and Asia. According to a 2015 study on how different European countries recorded and reported information on social determinants and risk factors among people with TB, some countries did or were considering expanding the collection of non-medical information, such as income or household size [19]. In the WHO European Region, and the post-Soviet states, there is high level of literacy, and informal health care providers are not plentiful, therefore these factors are not expected to influence access to TB diagnosis. Diagnostic delays in women have been observed in Ukraine [20] and in Georgia [21], however these studies did not include a qualitative component to learn more about the reasons for the delays directly from the people with TB. Our hypothesis was that factors related to gender norms contributed to delays in the diagnosis of TB in the countries of Eastern Europe and Central Asia (EECA), and we aimed to explore these factors by collecting and analyzing qualitative information. The information was collected as part of the broader TB communities, rights and gender assessments in Georgia, the Republic of Moldova, Kazakhstan and Tajikistan, which encompassed the topics of specific vulnerable to TB communities and populations, and the state of human rights and legal environments in relation to TB. The aim of the assessments, and of looking into gender-related factors was to build evidence, and inform improvements in TB programming and interventions.

## Methods

Retrospective information was collected through in-depth interviews and focus group discussions with people with a history of TB. Purposive sampling was used: respondents were first selected based on convenience (they were already clients of non-governmental organizations), and were available for an interview; a part of the respondents was randomly selected from the TB registries of patients and were available/willing to participate. Focus groups participants were recruited based on common characteristics. Additionally, for the interviews, a snowball technique was used where respondents could suggest other respondents (with a history of TB) to be interviewed. All eligible respondents were 18 years or older, provided written informed consent for their data to be included in the research process and met the criteria of having had TB no longer than five years prior to the date of data collection. Ethical permissions from the respective authorities to conduct the studies were obtained by all in-country teams.

Interview guides and focus group guides were based on a qualitative research tool developed by the Stop TB Partnership, which could be found at stoptb.org website. The tool was centered around the experiences of current and former TB patients, along the pathway of TB journey, starting with recognizing TB symptoms, and explored the themes of gender perceptions, vulnerability to TB, access to healthcare, diagnosis and treatment, quality of assistance received, and how to build a gender-sensitive TB response. The questions were adjusted to suit each country’s circumstances. The information was collected with the help of the affected communities, i.e., people with a history of TB disease were attracted, as much as it was feasible, to undergo training and subsequently carry out interviews or facilitate focus group discussions. Findings in each country were validated by means of workshops with the representatives of the communities affected by TB, and other stakeholders engaged in TB response.

In total there were 86 in-depth interview respondents and 21 focus groups (227 participants). Except for Kazakhstan, where there was an equal number of men and women among the respondents, in other countries there were more men; gender break-down of the respondents per country is in Table 1. The interviews and focus group discussions were recorded by all teams, some countries had them transcribed, others opted for extensive summaries, which were prepared by the interviewers immediately after each interview/focus group discussion, according to a pre-defined format.

**Table 1:** Gender break-down of the respondents per country

Country	Number of in-depth interviews	Gender break-down of the in-depth interview respondents			Number of focus groups with people with TB	Gender break-down of the focus groups’ participants			
		women	men	gender-diverse		women	men	gender-diverse	total
Georgia	30	33.3%	60%	6.7%	2 (1 men only and 1 women only)	6	10	0	16
Kazakhstan	30	50%	50%	0%	4 (2 men only and 2 women only)	22	22	0	44
Republic of Moldova	6	40%	60%	0	4 (2 men only, 1 women only and 1 mixed)	9	18	0	27
Tajikistan	20	40%	60%	0	11	80	60	00	140
Total number of respondents	86								227

Data were analyzed by the collaborating authors and their teams according to three pre-defined stages: symptom recognition, health care seeking and obtaining a correct diagnosis. Gender-related factors and barriers encountered by people with TB at each stage were noted by the interview and focus group note takers who were provided with a form to categorize information for ease of analyses.

Details about the methods based on the Consolidated criteria for Reporting Qualitative research (COREQ) Checklist are given in Table 2.

**Table 2:** Detailed methods based on COREQ Checklist

Information about the interviewer/focus group facilitator	Georgia	Kazakhstan	Republic of Moldova	Tajikistan
Domain 1: Research team and reflexivity				
Which author/s conducted the interview or focus group?	The co-author participated in the interviews and focus groups.	Interviews and focus groups were conducted by two contracted data collectors from the affected communities.	Interviews and focus groups were conducted by 12 contracted data collectors, mainly from the affected communities.	The co-author participated in the interviews and focus groups.
Did the gender of the interviewers match the gender of the interviewees?	No.	No, the two persons conducting interviews and facilitating focus groups were women.	No, all interviews were conducted by women.	Yes.
Where focus groups were not mixed, did the gender of the focus group facilitators match the gender of the focus group participants?	No.	No.	No, most moderators in focus groups were women.	Yes.
What experience and training did the interviewers have?	Interviewers were social workers, peer-educators with experience of working with TB key populations and conducting interviews. No extra training was provided. Interviewers belonged to the populations affected by TB. None of the interviewers had a history of TB.	One day training on interviewing and focus group facilitation was provided. The interviewers were employed by non-governmental organizations (NGOs) active in the field of TB.	One day training on interviewing and focus group facilitation was provided. The interviewers were employed by NGOs active in the field of TB, persons with a history of TB.	Interviewers were gender specialists, with experience in interviewing.
What experience and training did focus group facilitators have?	Facilitators were social workers, peer-educators with experience of working with TB risk groups and conducting focus groups.	One day training on interviewing and focus group facilitation was provided. Both female facilitators had personal experience of TB.	One day training on interviewing and focus group facilitation was provided. The facilitators were employed by NGOs active in the field of TB, including persons with a history of TB.	Facilitators received training. Skilled and experienced facilitators were selected for focus group discussions.
*Relationship with participants*				
Relationship established: Was a relationship established prior to study commencement?	Some of the respondents were beneficiaries of the NGOs that employed the interviewers.	Some participants had pre-established relations with the facilitator.	Some of the respondents were beneficiaries of the NGOs that employed the interviewers.	No.
Participant knowledge of the interviewer: What did the participants know about the researcher.	Participants did not have specific knowledge about the interviewer.	Participants knew that the interviewer had a history of TB.	Participants did not have specific knowledge about the interviewer.	Participants did not have specific knowledge about the interviewer.
Other characteristics about the inter viewer/facilitator.	None.	None.	None.	None.
Domain 2: Study design				
Theoretical framework	Barriers were analyzed along the stages that a person with TB goes through. The stage of diagnosis is preceded by the stage of symptoms recognition and the stage of seeking health care.			
Methodological orientation	Discourse analysis.			
*Participant selection*				
Sampling: How were participants selected?	Participants were selected on purposive basis: some from the TB registry and some were beneficiaries the NGOs or were recommended by other respondents based on the “snowball method”.	Participants were selected on purposive basis: some from the TB registry and some were beneficiaries the NGOs or were recommended by other respondents based on the “snowball method”.	Purposive and convenience methods used: some based on the NGOs territorial coverage: some from the TB registry.	Participants were selected on purposive basis: for focus groups by the NGOs that provided them with services and for the interviews - from on the TB registry to have equal numbers of men and women and those who were still on anti-TB treatment or had completed treatment by the time of the interview.
Method of approach: How were participants approached?	In case the participant was a beneficiary, they were approached face-to-face while receiving service at NGO premises grounds or were contacted by phone. Participants proposed based on TB registry were contacted by phone.	Face-to-face.	The recruitment of study participants was led by the NGOs using mostly telephone and face – to – face method of approach.	By telephone, email and personally through NGOs and TB doctors.
Sample size interviews	30	30	6	20
Number of focus groups and (number of participants)	2 (16)	4 (44)	4 (27)	11 (140)
Non-participation: How many people refused to participate or dropped out? Reasons?	Approximately one third, the main reason for non-participation was not wanting to hear about the disease again.	Unidentified number of people refused, women mostly referred to lack of time and the need to take care of children.	Approximately one third, due to various reasons such as personal reasons or competing priorities.	Several women with TB refused to participate because of fear of stigmatization.
*Setting*				
Setting of data collection: Where was the data collected?	At NGO premises.	Interviews were at TB clinic, HIV Center or NGO premises. Focus groups were also conducted at the locations convenient for the focus group participants.	At participants’ homes, NGO premises or TB facilities.	At NGO premises or at TB clinics.
Presence of nonparticipants: Was anyone else present besides the participants and interviewer/ facilitator and note taker?	No.	No.	No.	No.
Description of sample	People affected by TB/people with a history of TB.			
Dates of data collection	November – December 2019.	November 2019 – January 2020.	November – December 2021.	December 2019 – January 2020.
*Data collection*				
Interview guide	The interview guides were based on a generic tool, developed by the Stop TB Partnership; the interview and focus group guides were translated and adjusted to be relevant to the conductions of each participating country.			
Was the interview /focus group guide piloted in your country?	Yes.	Yes.	Yes.	Yes.
Repeat interviews	There were no repeat interviews.			
Recording: did the research use audio or visual recording to collect the data?	Audio recording was used.	Audio recording was used.	Audio and video recording were used.	Audio recording was used.
Field notes: Were field notes made during and/or after the interview or focus group?	Notes were taken by the assistant during the focus-groups. In case of interviews notes were made during the interview and after based on the recording.	Field notes taken during the interview or focus group.	Field notes taken during the interview or focus group.	Field notes taken during the interview or focus group.
Duration: What was the duration of the interviews?	Approximately 40 min.	Approximately 40 min.	Approximately 30–53 min.	Approximately 40–60 min.
Duration: What was the duration of the focus group?	Approximately 70 min.	Approximately up to 120 min.	Approximately 47–90 min.	6 Approximately 60–80 min.
Data saturation	Was not discussed, a fixed number of interviews/focus groups was pre-defined and carried out.			
Transcripts returned to participants	The transcripts were not returned to participants for comment and/or correction.			
Domain 3: analysis and findings				
Data analysis	Data was analyzed by the country-based collaborating authors and their teams according to pre-defined stages (symptom recognition, health care seeing and obtaining a correct diagnosis) and gender-related factors and barriers encountered by people with TB at each stage.			
Number of data coders	1	1	1	1
Description of the coding tree	Was not provided.			
Derivation of themes	The main themes were identified in advance, minor themes were derived from the data.			
Software	No qualitative data analysis software was used.			
Participant checking	Participants did not provide feedback on the findings.			
Reporting				
Quotations presented: were participant quotations presented to illustrate the themes/findings? Was each quotation identified?	Participant quotations were presented to illustrate the themes/findings; each quotation was not identified, except for gender and age.	Participant quotations were presented to illustrate the themes/findings; each quotation was not identified.	Participant quotations were presented to illustrate the themes/findings; each quotation was identified with an interviewer initials and the participant number for interviews, the location and date for focus groups.	Participant quotations were presented to illustrate the themes/findings; each quotation was not identified.
Data and findings consistent: was there consistency between the data presented and the findings?	Yes.	Yes.	Yes.	Yes.
Clarity of major themes: were major themes clearly presented in the findings?	Yes.	Yes.	Yes.	Yes.
Clarity of minor themes: is there a description of diverse cases or discussion of minor themes?	No.	No.	No.	No.

## Results

The results were structured around three main themes: gender-related barriers to symptom recognition, care seeking, and obtaining the correct diagnosis. The barriers were gender-related intra-family obligations and constraints, such as access to financial means and health-related decision making, concerns about losing work, and stigma.

We did not find differences between genders in the way participants recognized the symptoms of TB.

In terms of seeking TB diagnosis, women in Georgia, Kazakhstan and Tajikistan de-prioritized seeking diagnosis because of their lack of access to financial means, and obligations to care for the family and young children. A female respondent from Tajikistan reflected on the disadvantaged situation of daughters-in-law in the families, which was noted in Kazakhstan as well as in Tajikistan: “*We have such a mentality that a woman in the family does not have a voice, she is sick, but she cannot go to a medical institution, because of housework, childcare, or you need to get permission from your mother-in-law, demand money from your husband, considering all this, a woman does not go to a medical facility until she falls down.*” Women’s decision-making in relation to health was limited in Kazakhstan, Tajikistan and Georgia. Female-headed households in Tajikistan often struggled with poverty. In Georgia, women, especially in rural areas, had limited power over family budgets, were not be able to travel from a region to a big city/capital for a checkup and delayed diagnosis due to challenges in covering out-of-pocket payments.

In all four countries, traditionally carrying the weight of economically supporting the family, men tended to take no action or postpone TB diagnosis. In Kazakhstan men tended to self-medicate first before coming for a diagnosis, which resulted in delays in diagnosis. In the Republic of Moldova there were perceptions among men that they must appear strong, and men mentioned disregarding TB symptoms: “*Men think they are strong, the woman is waiting for you to bring money, you don’t have time to think, you have to work*”. [FG8-B1] A respondent in Georgia pointed out: “*Having TB means that I am already limited in choosing a career, now I cannot do physical work, since sweating, lifting heavy things or working in high humidity is not recommended. Also, why would an employer choose me, if there are people who are healthy and with no history of [TB] illness*”. In Tajikistan married men who work abroad make money remittances which are usually received not by their wives, but the (male) relatives on the husband’s side. This further contributes to women’s financial dependence and implicitly their freedom to seek medical care.

In Kazakhstan and Tajikistan women experienced stigma more often than men, and it is a deterrent factor to seeking healthcare. There is fear of divorce for married women while for the unmarried women a TB diagnosis means much worse prospects when finding a partner. According to a Tajik female respondent: “*We hid from everyone, neighbors and even relatives, that I had TB, because we were afraid that we would be rejected, no one would propose to me, and I would not be able to get married*”. A participant from Kazakhstan shared: “*I told [about TB] to my relatives, mother, sisters, brothers. My husband was told by the doctors. While there were only suspicions of TB, I told him myself, he was with me until they found it was TB for sure, he immediately began to move away from me and subsequently stopped all relations with me. After hospitalization, he visited me two times in the beginning. Then he did not even allow me to come home, did not let me into the house*” (woman, age 40). In Georgia too, women diagnosed with TB might get divorced by their husbands or lose prospects to get married. On the contrary, in Moldova the fear and shame of being associated with a socially disadvantaged group, was higher among men; two male respondents from a focus group mentioned: “*When people know that you once got sick they are very careful, no one wants to interact [with you], so they [people with TB] isolate themselves. […] They look at us as if we are the 4th, the 5th category, we are condemned plus have a disease*. [FG7-B1] *- Yes, that’s right, many are ashamed that they might have this disease and do not want to go look timely for the treatment and so they get sick from one another.”* [FG6-B7].

Georgia was able to collect information from gender-diverse people and Moldova could collect perceptions about this group. In Moldova a respondent commented regarding gender-diverse people that: “*Their way of life weakens their body and tuberculosis and other diseases can also lead to weak immunity*”, while others showed negative and stigmatizing attitudes towards gender-diverse people: “*They don’t make sense, what can we say to such peopl*e?” [FG1-F5] or “*Yes, their psyche is really sick*.” [FG8-F5]. In Georgia gender-diverse persons flagged stigmatization at medical institutions by health service providers, a transgender woman shared: “*When I went for a check-up at a primary healthcare facility, I heard the nurses giggling, they were hesitant to come near and would ask questions in a sarcastic manner regarding my recent “behaviors*”.

The last main theme of gender factors in obtaining the correct diagnosis came forth only in Georgia’s respondents and to some extent in Kazakhstan. A female respondent from Kazakhstan told: “*I asked in the summer to write a referral for X-ray, but they did not refer to this type of diagnosis, confusing the symptoms of TB with osteochondrosis, neuralgia, even after I was in emergency room, they released me by prescribing a painkiller with diphenhydramine. […] Only when my husband intervened, then they took an X-ray and identified TB, and that after seven-eight months have passed since my first appeal and request to do an X-ray*.” In Georgia TB was considered to be a disease of men, and women might not have been referred to TB diagnosis right away: “*At the first visit the doctor did not refer me to check for tuberculosis, saying there was a low probability, considering I was a woman and probably had not engaged in TB risk behavior*” – (woman, age 42). Women also felt discomfort in producing sputum samples, especially in front of male doctors: “*This was an awkward process for me, the doctor was waiting for me to cough, and another patient was already in line, after explaining that I was shy, only then they gave me space*” – (woman, age 32).

## Discussion

Our findings come from the four countries within the WHO European Region, which have commonalities, all being part of the Soviet block in the past, but slightly differ from one another in terms of the extent of TB epidemics, as well as in manifestations of the cultural and gender norms. In line with our expectation, literacy and seeking care from informal health care providers did not surface as prominent factors that influenced access to TB diagnosis. However, other factors related to gender norms contributing to delays in the diagnosis received support and confirmed our hypothesis. We explored factors related to delaying diagnosis due to gender-related intra-family obligations and gender-related constraints, such as lack of agency, stigma and discrimination, and health system related delays in diagnosis.

In contrast to findings made in the Philippines [9] or India [10], in Kazakhstan men, rather than women, were found to self-medicate. More information is needed to understand the reasons for this, as our study could not explain it based on the available data. Similarly to the observations made elsewhere, women were delaying diagnosis primarily because of a lack of childcare, care for elderly family members, lack of access to finance and limited decision-making, while men’s delaying was due to concerns about losing work. In all cases, outreach case finding including screening and diagnosis in near-by convenient locations, open outside of regular facility hours, and reduction of out-of-pocket payments, would facilitate access.

Stigma and confidentiality concern of women of marriageable age were pronounced among respondents from Kazakhstan and Tajikistan and echo the fears of women with TB in other countries. In contrast, in the Republic of Moldova men experienced stronger stigmatization which may have been associated with their inaction upon recognizing the symptoms of TB and delays in seeking diagnosis. Interestingly, the Republic of Moldova ris 28th in the 2021 Global Gender Gap Index [22] rankings, while Georgia, Kazakhstan and Tajikistan occupy respectively 62nd, 47th and 125th positions. It is possible that the more disadvantaged positions of women in Georgia, Kazakhstan and Tajikistan, are reflected in stronger perceived TB stigma in women in these three countries. Women in Georgia, Kazakhstan and Tajikistan may be feeling more stigmatized and discriminated as a result of TB than do women in the Republic of Moldova, where there is higher level of gender equity.

Information from and about gender-diverse people was scarce and gender-diverse respondents were few and only in Georgia. Nonetheless, there were indications in Georgia and in the Republic of Moldova that point to misconceptions held by other people with TB about gender-diverse people, and, in line with existing literature, evidence of gender-diverse people experiencing stigmatizing attitudes in health care settings. This implies the need to continue raising awareness among health providers about patients’ rights, including the right to confidentiality and the right to be free from discrimination regardless of their gender.

Findings from Georgia and Kazakhstan corroborate failures of health care providers to identify presumptive TB in women, which is in line with findings in several other countries. Although we did not find differences between genders in the way the study participants recognized the symptoms of TB, it is likely that health care providers suspected TB less often in women. This points to a need to improve awareness among health care providers about gender-related risks of TB and differences in the presentation and symptoms of TB. Women’s risk of TB is often related to care-giving functions when they tend for family and relatives with TB. Doing so before the sick family members are diagnosed and put on effective treatment means care-giving women get exposed to TB infection.

Our findings yield new evidence of gender-related barriers to TB diagnosis in the countries of EECA, especially among the gender-diverse people. While there are specifics that need to be reflected in each country’s TB response policies and programming, other findings confirm similarities with other countries and regions. Including social determinants in regional surveillance would help study the determinants further, raise awareness about their impact, and foster multi-sectoral response needed to address them [19]. Stigma is one example of an important determinant that could be monitored at the level of the countries and the EECA region. The 2022 “Tuberculosis surveillance and monitoring in Europe” report already underlines the role of stigma and discusses TB diagnostic delays in the context of COVID-19 [23].

There were several limitations of the study. First, the level of skills and experience of the interviewers and focus group facilitators varied, however there was always an effort to engage people with a personal experience of TB or persons from the TB affected communities. Second, because in each country the topic of gender and TB was a part of a larger assessments, covering other areas, there was not always a possibility to obtain rich information that could help understand some of the aspects of the barriers or explain reasons for certain choices or behaviors of the respondents. Third, it was not always possible to match the gender of the interviewer to the gender of the respondents and the gender of the focus group facilitator to the gender of the participants of the same-gender focus groups.

## Conclusion

This paper adds to the still limited body of updated evidence of gender-related barriers to the diagnosis of TB, a disease that affects millions of people yearly, and predominantly in low- and middle-income countries. These countries frequently struggle with gender inequalities and gender-based discrimination. Our findings highlight that there are gender-related as well as health system-related barriers to health care seeking and TB diagnosis that affect differently women, men and gender-diverse persons in the countries of the EECA Region. Barriers for women include stigma, and social reproduction responsibilities, such as caring for the children or elderly. While women struggle to access health care because they are financially dependent, men postpone a visit to a health care provider because they cannot miss work. Men may be afraid to lose their work, especially when they are the primary wage-earners and if the information about them having TB is not kept confidential, it may induce stigma, discrimination, and dismissal from work. In one country men flagged stigma and discrimination more often than did women. Stigma and discrimination limit access to health and TB diagnosis of gender-diverse people. Finally, the diagnosis of TB in women needs to receive more attention from health care providers, as failure to “think TB” may lead to delays in their diagnosis.

## Data Availability

The datasets generated and/or analysed during the current study are mostly comprised of interview records and are not publicly available in order to protect respondents’ confidentiality. Any data requests should be addressed to N. Turusbekova.

## Declarations

Authors confirm that all methods were carried out in accordance with relevant guidelines and regulations or Declaration of Helsinki.

Georgia: Institutional Review Board for National Center for Disease Control and Public Health / IRB 00002150;

Kazakhstan: Local Ethical Commission of the Asfendiyarov Kazakh National Medical University.

Tajikistan: Committee for Biomedical Ethics, Ministry of Health and Social Protection, Tajikistan, Academy of Medical Sciences.

The Republic of Moldova: National Committee for Ethical Expertise of Clinical trial, Ministry of Health, Labor and Social Protection of the Republic of Moldova.

## List of abbreviations

COREQ: - Consolidated criteria for Reporting Qualitative research
EECA: – Eastern Europe and Central Asia
HIV: - Human Immunodeficiency Virus
NGOs: - Non-governmental Organizations
TB: – Tuberculosis
WHO: – World Health Organization
